# Editorial: Advances in intra-abdominal infection

**DOI:** 10.3389/fmed.2025.1740463

**Published:** 2025-11-24

**Authors:** Feng Guo

**Affiliations:** 1 Department of Critical Care Medicine, Sir Run Run Shaw Hospital, Zhejiang University School of Medicine, Hangzhou, China; 2 Zhejiang Key Laboratory of Precise Diagnosis and Treatment of Abdominal Infection, Sir Run Run Shaw Hospital, School of Medicine, Zhejiang University, Hangzhou, China

**Keywords:** intra-abdominal infection, risk factor identification, antimicrobial therapy, nursing interventions, treatment strategies

Intra-abdominal infection (IAI) is a common yet severe clinical complication, encompassing conditions such as postoperative intra-abdominal infection (PIAI), liver abscess, and infection-related complications arising from interventional procedures (1, 2). It is associated with high morbidity and mortality rates, significantly impacting patient prognosis. Recent years have witnessed substantial progress in the identification of risk factors, development of predictive models, analysis of microbiological characteristics, optimization of treatment strategies, and implementation of nursing interventions for IAI, providing crucial guidance for clinical practice (3–5).

## Risk factor identification and early prediction model development

The accurate identification of high-risk patients and early warning of IAI are paramount for improving outcomes. A study by Ding et al. investigating liver abscess following thermal ablative therapy for liver cancer identified diabetes mellitus (OR = 3.215), a history of abdominal surgery (OR = 2.810), biliary tract disease (OR = 18.832, demonstrating the strongest correlation), and elevated alkaline phosphatase (ALP) levels (OR = 1.010) as independent risk factors. Biliary tract disease notably elevates infection risk through mechanisms involving bacterial contamination and bile stasis.

In patients undergoing gastrointestinal tumor surgery, PIAI represents a major obstacle to recovery. Researchers have integrated serum and drainage fluid (DF) biomarkers to develop nomogram models for predicting PIAI on postoperative day (POD) 1 and POD 3. The POD 1 model incorporates five indicators: Nutrition Risk Screening (NRS2002) score, POD1 C-reactive protein (CRP), POD1 interleukin-6 (IL-6), POD1 DF total protein (TP), and POD1 DF lactate dehydrogenase (LDH). The POD 3 model comprises four indicators: NRS2002 score, POD1 DF LDH, POD3 CRP, and POD3 DF LDH. Both models demonstrated excellent predictive performance in the validation cohort, with area under the curve (AUC) values of 0.958 (POD1) and 0.951 (POD3), enabling clinicians to identify high-risk patients at an early stage (Zhou et al.).

Studies have confirmed that an NRS2002 score ≥3 (indicating nutritional risk) and DF LDH levels (reflecting local tissue damage) serve as key predictors for PIAI. The combination of systemic inflammatory markers and local injury indicators enhances diagnostic accuracy (Zhou et al.).

## Microbiological characteristics and optimization of antimicrobial therapy

Elucidating the microbiological profile of IAI is essential for guiding appropriate antimicrobial use. Microbiological analysis of liver abscesses following thermal ablation for liver cancer revealed that Gram-negative bacteria accounted for 75.6% of 78 pathogenic strains isolated, with Escherichia coli (30.8%) and Klebsiella pneumoniae (20.5%) being the most prevalent. These two pathogens exhibited high susceptibility to amikacin, cefoxitin, imipenem, among others. However, the positive rates for extended-spectrum beta-lactamase (ESBL) production were 41.2% and 21.1%, respectively, underscoring the necessity for precise medication based on antimicrobial susceptibility testing results (Ding et al.).

In cases of ERCP-related Stapfer type IV injury complicated by infection, Morganella morganii, a member of the normal intestinal flora, was identified as a pathogenic bacterium. Its involvement in infection was associated with gas-driven translocation of intestinal flora post-injury, providing a novel etiological insight for the antimicrobial management of such complications (Liang et al.).

For immunocompromised patients with amoebic liver abscess (ALA) complicated by syphilis, serological testing (e.g., a positive indirect hemagglutination assay for amoebiasis) is critical for confirmation, even in the absence of positive pus cultures. Empirical therapy with metronidazole combined with cephalosporins, supplemented by percutaneous catheter drainage (PCD), yielded favorable outcomes. This highlights the importance of a comprehensive assessment utilizing multi-dimensional diagnostic approaches (Yang et al.).

## Evolving treatment strategies for special infection types

Treatment strategies for various types of IAI have been further refined based on clinical evidence. For ERCP-related Stapfer type IV injury, the simple defect itself is not considered a “true perforation,” and conservative management (e.g., anti-infective therapy, gastrointestinal decompression) is feasible. However, when concomitant severe infection is present, clinicians must remain vigilant for complications such as cholecystitis. Intervention targeting the source of infection (e.g., percutaneous transhepatic gallbladder drainage for cholecystitis) proves more effective than focusing solely on the injury site (Liang et al.).

All patients diagnosed with liver abscess following thermal ablation for liver cancer underwent percutaneous puncture drainage. Three cases required surgical drainage due to the failure of percutaneous attempts, indicating that the choice of drainage modality should be tailored to the patient's specific condition (Ding et al.). Traditional ALA management primarily relies on metronidazole, with drainage indicated in only approximately 15% of cases. However, for immunocompromised patients with concomitant conditions like syphilis or hepatitis B, early PCD combined pharmacotherapy can rapidly alleviate symptoms and reduce the risk of abscess rupture, even in the absence of overt signs of perforation, thereby expanding the indications for drainage therapy (Yang et al.).

## The role of nursing interventions in IAI prevention

Nursing interventions play an increasingly vital role in reducing the incidence of IAI. For patients presenting with acute abdominal pain, a graded nursing intervention model based on the Emergency Severity Index (ESI)—stratifying patients into Levels I-V with corresponding assessment and intervention timeframes—significantly improved clinical outcomes. Compared to routine nursing care, the graded intervention model reduced the total complication rate to 2% (vs. 14% in the routine care group), shortened emergency care time (34.62 min. vs. 64.20 min.) and hospital stay (7.64 days vs. 14.78 days), and increased nursing satisfaction to 98% (compared to 86% in the routine care group) (Jin et al.). This model optimizes the diagnosis and treatment process through accurate triage and systematic assessment, offering a scalable nursing protocol for IAI prevention.

## Conclusion

Recent advancements in IAI research—encompassing precise risk prediction via multi-marker models, pathogen-directed individualized antimicrobial therapy, personalized treatment plans for special infections, and effective graded nursing interventions—have substantially enhanced the precision and efficacy of clinical management. Future research efforts should focus on multi-center validation and long-term outcome assessment to further refine these strategies and ultimately improve patient prognosis.
